# Effects of endurance training on spirometric parameters in male long-distance runners

**DOI:** 10.3389/fphys.2026.1862024

**Published:** 2026-09-25

**Authors:** Anna Nagórka-Bolek, Aleksandra Undrunas, Wojciech Ratkowski, Aleksandra Puch-Walczak, Łukasz Wierucki, Adam Wyszomirski, Krzysztof Kuziemski, Krzysztof Flis, Tomasz Niźnikowski, Tomasz Zdrojewski

**Affiliations:** 1 Department of Preventive Medicine and Education, Medical University of Gdansk, Gdańsk, Poland; 2 Department of Recreational Sports, Gdansk University of Physical Education and Sport, Gdańsk, Poland; 3 Department of Adult Neurology, Faculty of Medicine, Medical University of Gdańsk, Gdańsk, Poland; 4 Department of Pulmonology, University of Warmia and Mazury, Olsztyn, Poland; 5 Gymnastics Department, Faculty of Physical Education and Health in Biala Podlaska, Józef Piłsudski University of Physical Education in Warsaw, Biała Podlaska, Poland

**Keywords:** endurance training, marathon runners, pulmonary function, respiratory system, spirometry

## Abstract

**Objectives:**

The aim of this study was to compare spirometric parameters between recreational male marathon runners (MR) and a control group (CG) representative of the male population of the Pomerania region to assess the association between endurance training and pulmonary function.

**Methods:**

A total of 31 amateur MR and 82 men in the CG aged 35–66 years participated in this study. The CG represented the male population of the Pomerania region (Poland). The analysis included basic spirometric parameters: forced vital capacity (FVC), forced expiratory volume in 1 s (FEV1), Tiffeneau index (FEV1/FVC), and the potential confounding factors: body mass index (BMI), waist circumference (WC), waist-to-hip ratio (WHR), smoking status, and history of obstructive lung diseases.

**Results:**

Recreational MR demonstrated significantly higher FVC and FEV1 values than the CG, both in absolute values and as percentages of predicted values (*p* < 0.001). Mean FVC was 5.24 ± 0.68 L in MR and 4.58 ± 0.95 L in controls, whilst mean FEV1 was 4.14 ± 0.55 L and 3.55 ± 0.88 L, respectively. No significant differences were observed in the Tiffeneau index (FEV1/FVC), smoking status, or prevalence of obstructive lung diseases between the groups. Although anthropometric parameters differed between groups, they were not significantly associated with spirometric outcomes.

**Conclusions:**

Recreational male MR demonstrated significantly higher FVC and FEV1 values than controls from the general male population. No significant differences were observed in the prevalence of obstructive airway disorders. Because of the relatively small sample size and cross-sectional design, the findings should be interpreted with caution. Further longitudinal studies are needed to better clarify the relationship between endurance training and pulmonary function.

## Introduction

Physical activity and its impact on human health have been extensively investigated across many fields of medicine. Regular physical activity has been shown to improve mental health and reduce the risk of coronary artery disease, hypertension, diabetes, and certain malignancies, including colon and breast cancer (Erbs et al., 2006; Wolin et al., 2009; Sieverdes et al., 2011; Aune et al., 2015; Herting and Chu, 2017; Maher et al., 2017; Mikkelsen et al., 2017; White et al., 2017; Rezende et al., 2018; Winzer et al., 2018). In addition, it contributes to the prevention of osteoporosis and falls in older adults and, most importantly, is associated with a reduced risk of premature all-cause mortality (Carter et al., 2001; Warburton et al., 2006; Troy et al., 2018).

Nowadays, participation in sports has become a routine part of daily life for millions of people worldwide. Running is one of the most popular forms of physical activity and is considered one of the most accessible leisure-time sports due to its minimal equipment and training requirements. It can be practised regardless of time or location, making it a convenient and natural form of exercise. Consequently, marathon running, despite its demanding nature, has gained increasing popularity since the first non-Olympic marathon race organised in Boston in 1897 (Knechtle et al., 2018).

Scientific interest in running has been driven largely by its potential health benefits, particularly with regard to cardiovascular fitness and longevity. Previous studies have demonstrated that runners have a 25%–40% lower risk of premature mortality and may live approximately 3 years longer than non-runners (Lee et al., 2017).

However, the effects of endurance running on the respiratory system remain insufficiently investigated. Available studies have focused primarily on elite athletes (Mazic et al., 2015), the prevalence of asthma symptoms amongst athletes (Kuziemski et al., 2009), comparisons of spirometric parameters across different sports disciplines (Lazovic et al., 2015), or changes in pulmonary function immediately after exercise (Miles et al., 1983). Spirometry has also been used to evaluate the association between physical activity and age-related decline in pulmonary function (Pelkonen et al., 2003). Nevertheless, current knowledge regarding the long-term effects of endurance training on respiratory function remains incomplete.

Therefore, the aim of the present study was to compare spirometric parameters between recreational male marathon runners (MR) and a control group (CG) representative of the general male population in order to assess the association between endurance training and pulmonary function.

## Materials and methods

The Marathon Project was conducted in the Pomerania region (Poland) at the Medical University of Gdansk and the University of Physical Education and Sport. It included a group of 31 men, amateur MR aged 35–66 years, volunteers invited to the study by the University of Physical Education and Sport in Gdansk. Participants were required to have completed at least one official marathon race (42.195 km) and to be actively engaged in long-distance running training. No women volunteered for the study; therefore, the study population consisted exclusively of men. The CG consisted of men selected from the *HF-Pomorskie* population study and served as a reference sample of the male population of the Pomerania region. Detailed methodology of *HF-Pomorskie* and selection of the group was described in a separate publication (Puch-Walczak et al., 2018). Among 700 individuals, the CG was selected to match the MR group with respect to age and sex distribution. The mean age did not differ significantly between the MR and CG groups (50.0 vs. 54.5 years, *p* > 0.05).

A questionnaire developed by the researchers was used to collect data on the runners’ physical activity and medical history, with particular emphasis on pulmonary diseases, current respiratory symptoms, and medication use. The questionnaire consisted of 49 items, including 45 closed-ended and 4 open-ended questions.

The examination included anthropometric measurements, non-invasive blood pressure measurement, spirometry, electrocardiography, echocardiography, and blood and urine tests to assess the general health status of the participants.

Quality-assured diagnostic spirometry was performed by an experienced technician under physician supervision in accordance with American Thoracic Society/European Respiratory Society (ATS/ERS) guidelines, thereby ensuring reproducibility of the results. First, the equipment (Spirometer Jaeger Masterscreen-Pneumo, Germany) passed a daily calibration prior to patient testing. The system was allowed to warm up for 10 min, after which the filter was installed and a 3-L syringe was attached to the sensor using an appropriate cone adapter. A 3-L volume was then injected into the spirometer at three different flow rates to assess device linearity. The measured volume fell within ±3.0% of the 3 L (between 2.91 and 3.09 L), in accordance with the standards of ATS/ERS. The spirometry protocol included static (slow) spirometry performed during calm breathing to assess lung volumes and capacities, as well as dynamic spirometry measuring airflow and volume during forced maximal respiratory manoeuvres with simultaneous generation of flow-volume curves. Spirometric results were subsequently evaluated and classified as (1) normal ventilatory function, (2) obstructive ventilatory abnormalities requiring bronchodilator reversibility testing, or (3) probable restrictive abnormalities requiring confirmation by whole-body plethysmography. The following spirometric indices were assessed: forced expiratory volume in 1 s (FEV1), vital capacity (VC), forced vital capacity (FVC), Tiffeneau ratio (FEV1/FVC), peak expiratory flow (PEF), maximal expiratory flow at 50% of FVC remaining to be exhaled (MEF50), maximal mid-expiratory flow between 25% and 75% of FVC (MEF25–75), forced expiratory time (FET), and inspiratory capacity (IC).

Although all standard spirometric parameters were measured, the present analysis focused primarily on FVC, FEV1, and the Tiffeneau ratio (FEV1/FVC). These parameters were analysed as absolute values, percentages of predicted values, and percentile distributions. According to the Global Initiative for Chronic Obstructive Lung Disease (GOLD) criteria, an FEV1/FVC ratio below 0.70 was used as the cutoff value for airway obstruction (Agustí et al., 2023). The age, sex, ethnic origin, and smoking status as the main determinants for the predicted values of spirometric measurement in adults were entered into the spirometer software database.

In the statistical analysis, Fisher’s exact test, Pearson’s chi-squared test, Student’s *t*-test, Wilcoxon rank-sum test, and Shapiro–Wilk test were used. Statistical significance was determined at *p* < 0.05. In the analysis of data with non-normal distributions, the median was used as the preferred measure of central tendency, because the median, unlike the mean, is less sensitive to outliers, so they do not distort the result. Data analysis was performed using R statistical software. The study protocol was approved by the Local Ethics Committee under reference number KB-27/17.

## Results

### Participant characteristics and training history

According to the questionnaire data, the MR group had been regularly engaged in long-distance running for a median of 8 years (**Table 1**). Participants continued regular running training immediately prior to the study and ran a median distance of 165 km during the 3 months preceding the examination, either individually or as part of amateur running groups. The runners had previously completed a median of 13 marathons, with the best recorded marathon time being 3:26:27.

**Table 1:** Physical activity in marathon runners.

Parameter regarding physical activity	Median (IQR)	Median 95% CI
Time of regular running training (years)	8 (5.8,12.8)	(6.5, 12.5)
Distance running up to 3 months before the research (km)	165 (117.5, 212.0)	(140.0, 242.5)
Marathons finished in the past	13.0 (6.8, 23.3)	(9.5, 27.0)
Best result in official marathon race (hours: minutes: seconds)	03:26:27 (02:57:54, 03:37:42)	(03:11:38, 03:37:48)

IQR, interquartile range; 95% CI, 95% confidence interval.

The mean age did not differ significantly between the MR and CG groups (50.0 vs. 54.5 years, *p* > 0.05). MR had significantly lower body mass index (BMI) values than CG. Overall, excessive body weight was observed in 58.0% of MR and 70.5% of CG participants. A higher proportion of MR were classified as overweight (54.8%); however, only one runner was obese (3.3%).

In the CG group, 38.7% of participants were overweight and 31.8% were obese. CG demonstrated higher waist circumference (WC) and waist-to-hip ratio (WHR) values than MR. In addition, WC ≥ 94 cm and WC ≥ 102 cm, indicating abdominal obesity, were observed more frequently in CG (**Table 2**). Statistical analysis showed that BMI, WC, and WHR were not significantly associated with spirometric outcomes.

**Table 2:** Comparison of marathon runners and the control group with respect to age, anthropometric parameters, and smoking status.

Parameter	MR	CG	p-value
Age (years)			ns
Median (IQR)	50.0 (45.5, 54.5)	54.5 (46.0, 62.0)	
Median 95% CI	(47.5, 53.0)	(52.0, 56.0)	
BMI			0.007
Median (IQR)	25.6 (23.3, 26.9)	27.6 (24.4, 30.7)	
Median 95% CI	(24.33, 26.33)	(26.72, 28.94)	
WC (cm)			< 0.001
Median (IQR)	90.0 (85.0, 94.0)	99.0 (89.0, 108.0)	
Median 95% CI	(87.0, 92.0)	(96.0, 102.0)	
WC ≥ 94 cm			0.003
(% N)	29	60.5	
WC ≥ 102 cm			< 0.001
(% N)	0	40.7	
WHR			< 0.001
Median (IQR)	0.91 (0.88, 0.96)	0.98 (0.97, 1.02)	
Median 95% CI	(0.90, 0.94)	(0.98, 1.0)	
Smoking status			
Current smokers (% N)	9.7	23.2	ns
Current non-smokers (% N)	90.3	76.8	ns
Smokers in the past (% N)	32.3	28.0	ns

MR, marathon runners; CG, control group; BMI, body mass index; WC, waist circumference; WHR, waist-to-hip ratio; IQR, interquartile range; 95% CI, 95% confidence interval; ns, non-significant.

### Respiratory symptoms and medical history

No significant differences were observed in current or past smoking status between the groups (**Table 2**). Joint injuries were the most commonly reported medical condition amongst MR, whereas hypercholesterolaemia, arterial hypertension, and atherosclerosis were reported less frequently (**Table 3**). Two of the 31 runners had previously been diagnosed with asthma. None of the participants reported other chronic respiratory diseases, including chronic obstructive pulmonary disease, chronic bronchitis, or emphysema. The prevalence of obstructive pulmonary diseases did not differ significantly between MR and CG.

**Table 3:** Prevalence of reported medical conditions among marathon runners (MR).

Medical condition	N	Percentage of the MR (%)
Joint injuries	14	45.16
Hypercholesterolemia	8	25.81
Arterial hypertension	7	22.58
Atherosclerosis	4	12.91
Asthma	2	6.45
Diabetes	1	3.23
Neoplasm	1	3.23
Heart defect	1	3.23
Viral hepatitis type B	1	3.23
Myocardial infarction Coronary artery disease Heart failure Stroke Atrial fibrillation Atrial flutter Aortic aneurysm Chronic kidney disease Chronic obstructive pulmonary disease Chronic bronchitis Emphysema	None reported	

Regarding respiratory symptoms, two athletes reported a history of dyspnoea (**Table 4**). Both individuals had normal spirometry results. One participant associated dyspnoea with periods of increased physical activity, accompanied by palpitations and tachycardia; this individual also had a medical history of arterial hypertension and hypercholesterolaemia. One runner reported wheezing and had previously been diagnosed with asthma. Spirometric examination revealed an FEV1/FVC ratio below 0.70. Chest pain accompanied by palpitations and hot flushes was reported by one participant. No participant reported chronic cough or haemoptysis.

**Table 4:** Prevalence of reported symptoms among marathon runners (MR).

Symptom	N	Percentage of the MR (%)
Muscle or bone pain	7	22.58
Palpitations or irregular heartbeat	4	12.90
Dyspnoea	2	6.45
Wheezing	1	3.23
Hot flushes	1	3.23
Chest pains	1	3.23
Racing heartbeat	1	3.23
Haemoptysis	0	0.00
Cough lasting for over 8 weeks	0	0.00

### Spirometry findings

Although all mean spirometric values remained within the normal range, long-distance runners demonstrated significantly higher FVC and FEV1 values than CG, both in absolute terms and relative to predicted values (**Table 5**). In the MR group, FVC and FEV1 values exceeded 100% of predicted values. Moreover, FVC values exceeded the 95th percentile more frequently and were less likely to fall below the 5th percentile amongst MR compared with CG (**Table 6**). No significant differences were observed between the groups in the Tiffeneau ratio with respect to percentile distribution, absolute values, or predicted values. BMI, WC, WHR, and smoking status were not significantly associated with spirometric parameters.

**Table 5:** Spirometric parameters in marathon runners and the control group.

Spirometric parameters	MR	CG	p-value
FVC			
Best (L)			< 0.001
Mean ( ± SD)	5.24 ( ± 0.68)	4.58 ( ± 0.95)	
Mean 95% CI	(5.0, 5.49)	(4.37, 4.79)	
Median (IQR)	5.2 (4.84, 5.58)	4.62 (4.03, 5.18)	
Best/predicted value (%)*			< 0.001
Median (IQR)	113.3 (105.15, 121.55)	100.3 (93.08, 107.78)	
Median 95% CI	(108.85, 117.75)	(97.6, 102.95)	
FEV1			
Best (L)			< 0.001
Mean ( ± SD)	4.14 ( ± 0.55)	3.55 ( ± 0.88)	
Mean 95% CI	(3.94, 4.34)	(3.35, 3.74)	
Median (IQR)	4.01 (3.81, 4.52)	3.66 (2.96, 4.21)	
Best/predicted value (%)*			< 0.001
Median (IQR)	112.5 (103, 119.75)	98.15 (86.88, 106.88)	
Median 95% CI	(106.6, 115.85)	(91.85, 99.8)	
FEV1/FVC			
Best (%)*			ns
Median (IQR)	77.19 (74.65, 80.85)	77.32 (70.33, 81.13)	
Median 95% CI	(75.61, 79.21)	(73.79, 78.0)	
Best/predicted value (%)			ns
Mean ( ± SD)	98.72 ( ± 6.78)	96.35 (11.55)	
Mean 95% CI	(96.23, 101.2)	(93.81, 98.89)	
Median (IQR)	100.3 (95.35, 103.2)	98.65 (90.15, 105.5)	

MR, marathon runners; CG, control group; FEV1, forced expiratory volume in 1 second; FVC, forced vital capacity; FEV1/FVC, Tiffeneau index; SD, standard deviation; IQR, interquartile range; 95% CI, 95% confidence interval; ns, non-significant; * non-normal distribution; the median was used as the preferred measure of central tendency.

**Table 6:** Spirometric parameters in percentile distribution.

Spirometric parameter in percentile distribution	MR (N, %N)	CG (N, %N)	p-value
FEV_1_		ns	
<5	0 (0.0%)	10 (12.2%)	
[5, 95]	27 (87.1%)	66 (80.5%)	
>95	4 (12.9%)	6 (7.3%)	
FVC		0.022	
<5	0 (0.0%)	4 (4.9%)	
[5, 95]	24 (77.4%)	73 (89.0%)	
>95	7 (22.6%)	5 (6.1%)	
FEV_1_/FVC		ns	
<5	2 (6.5%)	12 (14.6%)	
[5, 95]	28 (90.3%)	69 (84.1%)	
>95	1 (3.2%)	1 (1.2%)	

MR, marathon runners; CG, control group; FEV1, forced expiratory volume in 1 second; FVC, forced vital capacity; VC, vital capacity; FEV1/FVC, Tiffeneau index; ns, non-significant.

## Discussion

### Limitations

The present study included a group of recreational male long-distance runners who had been regularly training for many years, had repeatedly participated in marathon races, and remained physically active immediately prior to the examination. The inclusion of a CG representative of the general male population further strengthens the study.

Nevertheless, several limitations should be considered when interpreting the results. First, the study population was relatively small. Recruiting a larger cohort of participants meeting the inclusion criteria and willing to undergo a comprehensive multi-stage examination is challenging. In addition, the study included only men; therefore, the findings cannot be generalised to the entire population of MR.

Differences between the MR and CG groups were also observed with respect to anthropometric parameters and smoking exposure expressed as pack-years. MR were significantly leaner and demonstrated a lower prevalence of abdominal obesity. Consequently, it remains unclear whether the observed differences in spirometric parameters resulted directly from endurance training or were partially associated with differences in body composition and smoking exposure. However, statistical analysis demonstrated no significant associations between BMI, WC, WHR, smoking status, pack-years, and spirometric outcomes, suggesting that these variables were unlikely to have substantially influenced the results.

### Comparison with previous studies

The beneficial effects of physical activity on respiratory function have previously been described in athletes participating in rowing, swimming, football, basketball, handball, water polo, and boxing (Miles et al., 1983; Pelkonen et al., 2003; Stanojevic et al., 2008; Kuziemski et al., 2009; Lazovic et al., 2015; Mazic et al., 2015; Rubini et al., 2017; Puch-Walczak et al., 2018; Agustí et al., 2023). However, data regarding amateur long-distance runners remain limited. The present findings complement the study by Mazic et al., in which elite athletes demonstrated higher FEV1, FVC, and VC values than control participants (Mazic et al., 2015).

Similar findings have been reported in studies involving young and elite athletes. Lazovic-Popovic et al. observed significant differences in dynamic pulmonary function parameters, including VC, FVC, FEV1, and FEV1/VC, in young athletes (Lazovic-Popovic et al., 2016). Other studies comparing different sports disciplines have also shown that athletes may demonstrate higher lung volumes and spirometric values than sedentary controls, although the magnitude and pattern of these differences appear to depend on the type of sport. In the present study, MR had higher FVC and FEV1 values, whereas no significant difference in the Tiffeneau ratio was observed. Previous studies have also shown that several weeks of intensive training may improve spirometric parameters, further supporting the relationship between physical activity and pulmonary function (Nourry et al., 2005; Khosravi et al., 2013; Rubini et al., 2017).

### Possible mechanisms

Regular physical activity may increase respiratory muscle strength and endurance, particularly of the diaphragm and intercostal muscles, thereby allowing greater lung expansion and more forceful expiration. Enhanced thoracic mobility and improved chest wall compliance may additionally contribute to larger ventilatory volumes. Furthermore, higher aerobic fitness has been associated with reduced airway resistance and improved ventilatory efficiency, which may explain the elevated FEV1 values observed in trained runners. Taken together, these physiological adaptations may contribute to the superior pulmonary function commonly observed in endurance athletes (Lazovic et al., 2015).

## Conclusion

Recreational male MR demonstrated higher FVC and FEV1 values than controls representative of the general male population, although mean spirometric values in both groups remained within the normal range. No significant differences were observed in the Tiffeneau ratio or in the prevalence of obstructive airway disorders between the groups.

These findings suggest that long-distance running may be associated with favourable pulmonary adaptations, potentially related to improved respiratory muscle performance and ventilatory efficiency. However, because of the relatively small sample size and cross-sectional design, the findings should be interpreted with caution.

Further longitudinal studies involving larger cohorts, including both male and female participants, are required to better clarify the relationship between endurance training and pulmonary function. The findings may have practical implications for sports medicine and respiratory health assessment by improving understanding of physiological respiratory adaptations associated with long-term endurance training.

## Data Availability

The raw data supporting the conclusions of this article will be made available by the authors, without undue reservation.
